# Prognostic Value of SII for Prediction of Pharmacological Cardioversion Success in Newly Diagnosed Atrial Fibrillation

**DOI:** 10.3390/jcm15041407

**Published:** 2026-02-11

**Authors:** Çetin Mirzaoğlu, Barış Karaca, Mehdi Karasu, Yücel Karaca, Özkan Yavçin, Mehmet Ali Gelen

**Affiliations:** 1 Department of Cardiology, Fethi Sekin Sehir Hastanesi, Elazıg 23270, Turkey; dr.cmirzaoglu@gmail.com (Ç.M.);; 2 Department of İnternal Medicine, Adıyaman Egitim ve Arastırma Hastanesi, Adıyaman 02040, Turkey; 3 Department of Cardiology, Adıyaman Egitim ve Arastırma Hastanesi, Adıyaman 02040, Turkey

**Keywords:** atrial fibrillation, pharmacological cardioversion, systemic immune-inflammation index, inflammation, amiodarone, rhythm control

## Abstract

**Background:** Pharmacological cardioversion (PC) with antiarrhythmic agents is a common initial rhythm control strategy in patients with new-onset atrial fibrillation (AF). However, predictive tools for estimating the likelihood of successful PC remain limited. The systemic immune-inflammation index (SII), a novel composite marker derived from neutrophil, lymphocyte, and platelet counts, may reflect atrial inflammatory burden and structural remodeling. This study aimed to investigate the prognostic value of SII in predicting pharmacological cardioversion success in patients with acute-onset symptomatic AF. **Methods:** This prospective observational study included patients with hemodynamically stable, new-onset symptomatic AF admitted since October 2025. All patients received intravenous amiodarone for pharmacological cardioversion. Baseline clinical, echocardiographic, and laboratory parameters were recorded. Patients were classified into cardioversion-success and non-response groups based on ECG-confirmed restoration of sinus rhythm. Logistic regression analyses were performed to identify independent predictors of rhythm control, and ROC curves were generated to determine predictive performance. **Results:** Among 95 patients (mean age 54.2 ± 9.8 years, 48.4% female), successful pharmacological cardioversion was achieved in 74.7%. Compared to the non-response group, the cardioversion-success group had significantly lower SII levels (*p* < 0.001) and left atrial volume index (LAVI, *p* < 0.001). Multivariate analysis identified both SII and LAVI as independent predictors of cardioversion success. Inverse correlations were observed between both SII (r = −0.419, *p* < 0.01) and LAVI (r = −0.567, *p* < 0.01) and rhythm control. The optimal SII cutoff of 645.16 predicted successful rhythm restoration with 75% sensitivity and 75% specificity (AUC: 0.803, 95% CI: 0.710–0.895). **Conclusions:** Higher SII levels were independently associated with lower rates of successful pharmacological cardioversion in patients with new-onset atrial fibrillation. Incorporating SII into routine assessment may enhance clinical decision-making and patient stratification for rhythm control strategies.

## 1. Introduction

Atrial fibrillation (AF) is the most common arrhythmia and is associated with increased morbidity, mortality, and healthcare costs [1].

The clinical burden of atrial fibrillation (AF) extends beyond its well-known association with systemic thromboembolism and adverse cardiovascular outcomes; it also significantly impairs patients’ overall quality of life (QoL) [2]. In symptomatic individuals, rhythm control—implemented alongside adequate rate management—primarily aims to alleviate symptoms and improve daily functioning [3]. Emerging evidence further suggests that an early and comprehensive rhythm-control strategy, incorporating antiarrhythmic medications (AADs), electrical cardioversion (EC), and catheter-based interventions such as pulmonary vein isolation, may contribute to better long-term outcomes when initiated during the initial phases of the disease [4].

Pharmacological cardioversion (PC) usually represents the first therapeutic step in rhythm restoration. This approach involves the oral or intravenous administration of AADs under continuous electrocardiographic monitoring with the goal of converting AF to sinus rhythm (SR) [5]. When successful, PC can terminate symptomatic AF episodes, enhance QoL, prevent unnecessary hospital admissions, and ultimately reduce healthcare utilization [6].

According to the current European Society of Cardiology (ESC) guidelines, both PC and EC are recommended as initial management options for patients presenting with recent-onset and symptomatic AF, provided that no contraindications exist [7]. Although EC achieves SR in approximately 90% of cases, its need for sedation or general anesthesia often makes pharmacological intervention the preferred first attempt in clinical practice [8]. The overall effectiveness of PC varies depending on patient characteristics and the selected AAD, but reported conversion rates generally fall within the range of 50% to 70% [9].

As the prevalence of atrial fibrillation (AF) continues to rise, along with its associated economic impact, a variety of clinical scoring systems have been introduced to estimate an individual patient’s risk for adverse outcomes such as thromboembolism, major bleeding, mortality, and treatment-related complications [10,11]. Despite these advances, no validated instrument currently exists to predict the likelihood of achieving successful pharmacological cardioversion in a patient presenting with symptomatic AF. Nevertheless, having access to such individualized prognostic information would be highly valuable in the clinical setting, where shared decision-making between physicians and patients is increasingly emphasized.

Accurate predictive tools can enhance this collaborative process by enabling patients to better understand the potential benefits and risks of available therapeutic strategies, as well as the probability of their success. This, in turn, may facilitate the selection of the most appropriate management approach tailored to their clinical profile and personal preferences.

In this context, the present study was designed to establish and validate a predictive model for the success of intravenous pharmacological cardioversion in individuals with hemodynamically stable, acute-onset symptomatic AF. The goal was to estimate each patient’s personalized probability of achieving sinus rhythm through drug-based cardioversion.

İnflammatory changes may link the etiology of AF, associated comorbidities and risk factors with electrical and structural cardiac remodeling, cardiac damage, myocardial fibrotic changes, microvascular dysfunction and altered reparative response [12].

The systemic immune-inflammation index (SII)—calculated using the formula *platelet count* × *neutrophil count*/*lymphocyte count*—was originally proposed as a composite marker reflecting the overall balance between host immune activation and inflammatory status [13]. This index offers a straightforward, inexpensive, and readily obtainable measure derived from routine complete blood count parameters, making it practical for widespread clinical use.

Although initially introduced as a general marker of systemic inflammation, the SII has since been evaluated in numerous clinical contexts. A growing body of evidence suggests that it may hold prognostic significance across a broad spectrum of diseases in which inflammation plays a central role in disease development or progression. These include cardiovascular disorders, malignant diseases, infectious conditions, and various chronic inflammatory states, where the SII has demonstrated potential in predicting clinical outcomes through diverse pathophysiological pathways.

The aim of this study is to investigate the predictive value of SII for the success of pharmacological cardioversion in new onset atrial fibrilation.

## 2. Method

This prospective observational study included all consecutive patients who presented to our emergency department or cardiology outpatient clinic with a first episode of atrial fibrillation (AF) beginning in October 2025. The diagnosis of AF was confirmed in each case by a standard 12-lead electrocardiogram (ECG) demonstrating an irregular rhythm without discrete P waves. Enrollment occurred after obtaining written informed consent from all participants.

Upon initial evaluation, a structured clinical assessment form was completed by the treating physician. This form captured vital parameters—heart rate, systolic and diastolic blood pressure, peripheral oxygen saturation—as well as a detailed characterization of AF-related symptoms (palpitations, dyspnea, chest discomfort, fatigue) and an estimation of symptom onset time. The type of AF (first-diagnosed, paroxysmal, or unknown duration) was classified according to European Society of Cardiology (ESC) criteria. All therapeutic interventions administered during the index visit, including intravenous rate-control agents, electrolyte replacement therapy, and pharmacological or electrical cardioversion attempts, were recorded in real time.

Comprehensive demographic and clinical information was also collected. This included age, sex, comorbidities (hypertension, diabetes mellitus, coronary artery disease, heart failure, etc.), concomitant medications, prior history of AF, and any previous attempts at electrical cardioversion. Stroke risk was assessed using the CHA_2_DS_2_-VASc score. Laboratory data captured at admission included complete blood count parameters, routine biochemical panel, renal and hepatic function tests, standard coagulation measurements, thyroid function tests, serum troponin levels, and N-terminal pro-brain natriuretic peptide (proBNP) values. All laboratory tests were performed using standardized assays in the hospital’s central laboratory.

### 2.1. Eligibility Criteria

Patients qualified for inclusion if they met all of the following conditions:

1. Hemodynamic stability at presentation (absence of shock, severe hypotension, or ongoing myocardial ischemia).
2. Symptomatic new-onset AF, with symptoms prompting medical evaluation.
3. Non-permanent AF documented since October 2025.
4. Ability to provide informed consent.

To ensure the reliability of clinical and laboratory-based predictive modeling, the following exclusion criteria were applied:

- Presence of permanent AF or long-standing persistent AF.
- Spontaneous conversion to sinus rhythm before pharmacological therapy.
- Immediate direct electrical cardioversion performed due to clinical indications.
- Laboratory evidence of abnormal thyroid function, elevated troponin, or proBNP levels above reference range, which could independently influence rhythm outcomes.
- Incomplete clinical or laboratory data.

**Figure 1:**
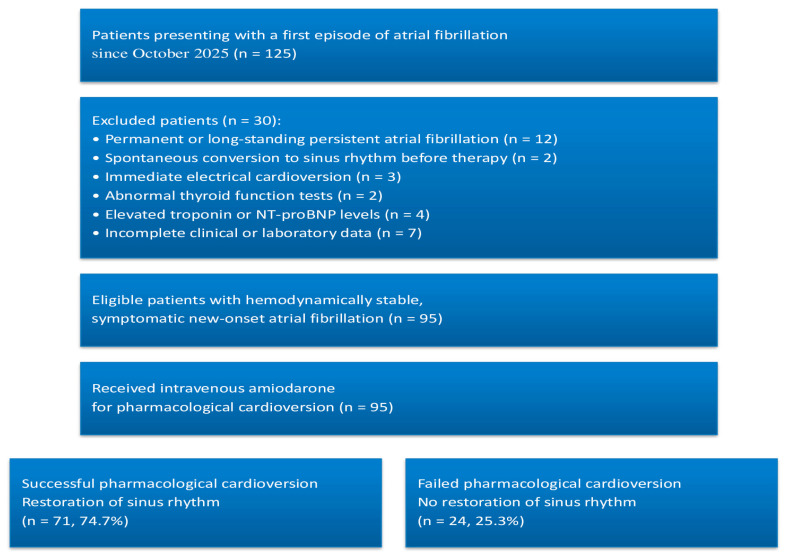
Patient Recruitment Flowchart. Flowchart illustrating patient screening, exclusion criteria, and final study population. After application of predefined exclusion criteria, 95 hemodynamically stable patients with symptomatic new-onset atrial fibrillation underwent pharmacological cardioversion with intravenous amiodarone and were classified according to cardioversion success.

A patient recruitment flowchart illustrating screening, exclusions, and the final study population was added (Figure 1).

### 2.2. Pharmacological Cardioversion Protocol

Pharmacological cardioversion (PC) was conducted using intravenous amiodarone as the standard agent, in accordance with institutional practice guidelines. The medication was administered under continuous ECG monitoring and routine hemodynamic surveillance. To avoid misclassification, therapies aimed solely at rate control—such as beta-blockers, calcium channel blockers, or digoxin—as well as electrolyte correction (e.g., magnesium or potassium supplementation) were not considered cardioversion attempts.

Successful pharmacological cardioversion was defined as documented restoration of sinus rhythm on a follow-up 12-lead ECG obtained after the completion of intravenous amiodarone infusion and without requiring electrical cardioversion. ECGs were interpreted independently by two cardiologists; discrepancies were resolved by consensus.

### 2.3. Data Management and Ethical Considerations

All clinical information was entered into a secure, password-protected electronic database accessible only to the study investigators. Data were routinely checked for completeness, internal consistency, and accuracy. The study protocol conformed to the principles of the Declaration of Helsinki and received approval from the institutional ethics committee of Fethi Sekin City Hospital (2 October 2025, 2025/16-15). Informed consent was obtained from all participants of the study. Patient confidentiality was preserved by assigning anonymized study identification numbers.

### 2.4. Statistical Analysis

Continuous variables were summarized either as mean ± standard deviation (SD) or as median values with their corresponding interquartile ranges, depending on data distribution. Categorical variables were reported as counts and percentages. Comparisons between the two study groups were performed using the independent Student’s *t*-test for normally distributed continuous variables, whereas the Mann–Whitney *U* test was applied when normality assumptions were not met. Categorical data were evaluated using either the chi-square test or Fisher’s exact test, as appropriate.

Univariate logistic regression analyses were first performed to identify variables associated with successful pharmacological cardioversion. Variables demonstrating statistical significance in univariate analyses or considered clinically relevant were subsequently entered into a multivariate logistic regression model. Multicollinearity among predictors was assessed using variance inflation factor (VIF) analysis, with no evidence of significant collinearity observed. Receiver operating characteristic (ROC) curve analysis was utilized to determine the optimal threshold of the inflammation index for predicting successful rhythm control. The cutoff point corresponding to the highest Youden index (sensitivity + specificity − 1) was selected as the optimal discriminatory value. The area under the ROC curve (AUC) was calculated, and pairwise comparisons were performed when necessary.

A two-tailed *p* value of <0.05 was considered indicative of statistical significance. All statistical procedures were carried out using SPSS Statistics software (version 26.0; IBM SPSS, Chicago, IL, USA).

## 3. Results

The study population had a mean age of 54.2 ± 9.8 years, and 48.4% of participants were female. Baseline demographic, clinical, laboratory, and echocardiographic characteristics are summarized in Table 1. There were no significant differences between patients who achieved successful rhythm control and those who did not with respect to age, CHA_2_DS_2_-VASc score, heart rate, left ventricular ejection fraction, serum creatinine, or hemoglobin levels.

Significant differences were observed in inflammatory, metabolic, and echocardiographic parameters between the two groups. Patients who failed to achieve rhythm control exhibited significantly higher white blood cell and neutrophil counts, higher left atrial volume index (LAVI), higher serum glucose levels, and elevated systemic immune–inflammation index (SII). In contrast, body surface area (BSA) and lymphocyte counts were significantly higher among patients who achieved successful rhythm control.

In univariate logistic regression analyses using standardized variables, higher LAVI (OR per 1 SD increase: 0.23, 95% CI: 0.12–0.45; *p* < 0.001), higher SII (OR: 0.35, 95% CI: 0.19–0.64; *p* = 0.001), higher white blood cell count (OR: 0.57, 95% CI: 0.35–0.94; *p* = 0.029), and higher serum glucose levels (OR: 0.63, 95% CI: 0.40–0.98; *p* = 0.042) were each associated with a lower likelihood of successful pharmacological cardioversion. Conversely, higher BSA was associated with an increased probability of rhythm control (OR: 1.80, 95% CI: 1.07–3.03; *p* = 0.027).

In the multivariate logistic regression model incorporating all standardized variables, only LAVI and SII remained independently associated with rhythm control. Each one standard deviation increase in LAVI was associated with an 82% reduction in the likelihood of successful cardioversion (OR: 0.18, 95% CI: 0.08–0.42; *p* < 0.001), while a one standard deviation increase in SII was associated with a 78% reduction in cardioversion success (OR: 0.22, 95% CI: 0.08–0.58; *p* = 0.003). White blood cell count, glucose level, and BSA were not independently associated with rhythm control in the adjusted model (Table 2).

**Figure 2:**
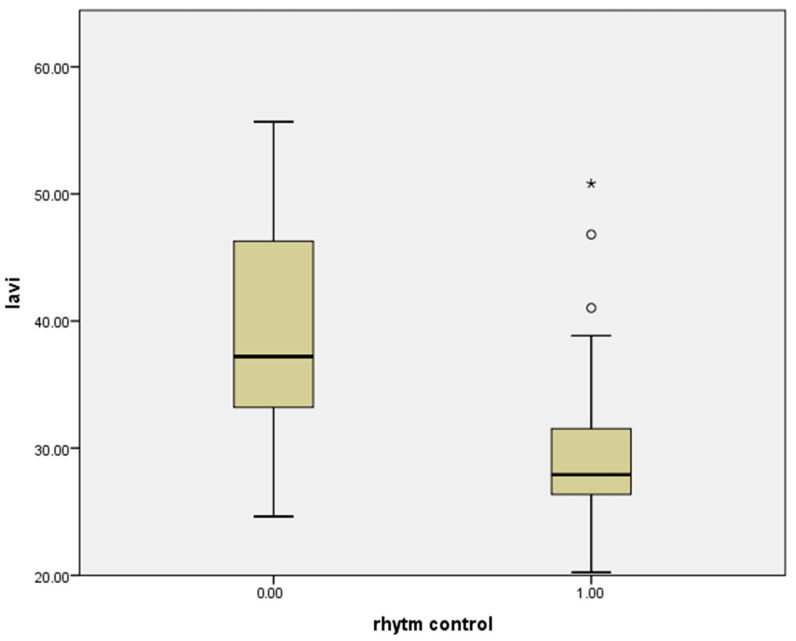
Correlation of LAVİ with pharmacological cardioversion success. * Correlation is significant at the 0.05 level (R: −0.567 *p* < 0.01).

**Figure 3:**
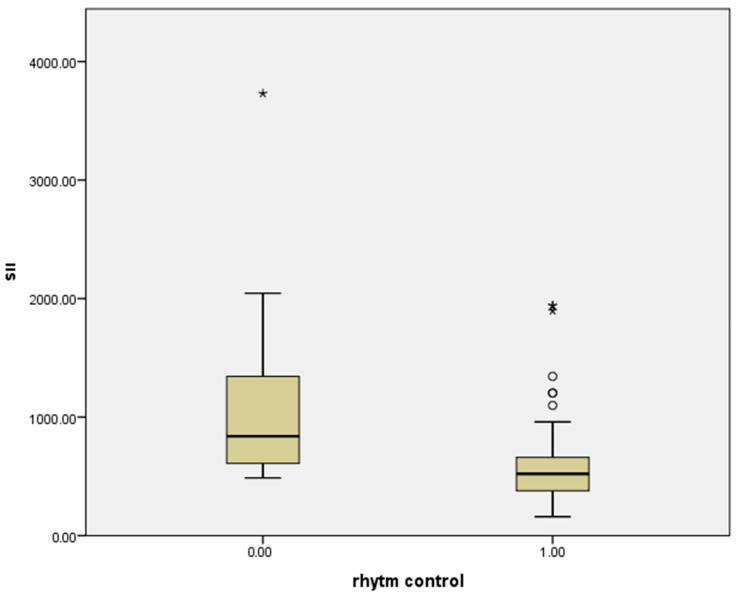
Correlation of SII with pharmacological cardioversion success. * Correlation is significant at the 0.05 level. ** Correlation is significant at the 0.01 level (R: −0.419 *p* < 0.01).

Correlation analysis demonstrated a significant inverse relationship between LAVI and rhythm control (r = −0.567, *p* < 0.01; Figure 2). Similarly, SII was inversely correlated with rhythm control (r = −0.419, *p* < 0.01; Figure 3).

**Figure 4:**
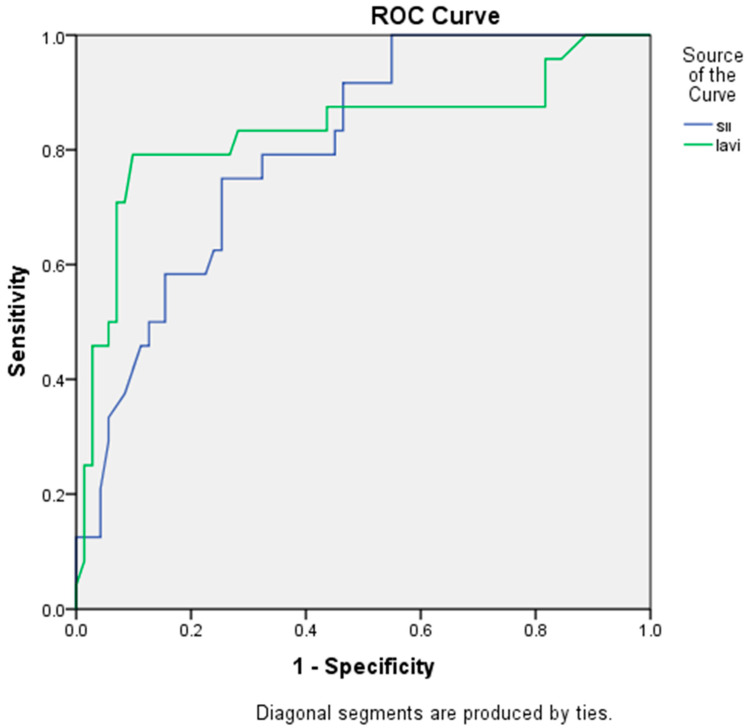
ROC analysis of SII for pharmacological cardioversion success. The optimal SII cutoff of 645.16 predicted successful rhythm control with 75% sensitivity and 75% specificity. SII AUC: 0.803(%95 CI 0.710–0.895 *p* < 0.01). LAVİ AUC: 0.833 (%95 CI 0.719–0.947 *p* < 0.01).

Receiver operating characteristic curve analysis comparing the discriminative performance of LAVI and SII is presented in Figure 4. LAVI demonstrated slightly superior predictive performance compared with SII (AUC = 0.833, 95% CI: 0.719–0.947; *p* < 0.01 vs. AUC = 0.803, 95% CI: 0.710–0.895; *p* < 0.01). An SII cut-off value of 645.16 predicted successful rhythm control with a sensitivity of 75% and a specificity of 75%.

## 4. Discussion

In this study, we demonstrated that the Systemic Immune-Inflammation Index (SII) is an independent predictor of successful pharmacological cardioversion in patients with newly diagnosed atrial fibrillation (AF). Elevated SII levels were significantly associated with failure to achieve sinus rhythm following amiodarone administration. To our knowledge, this is among the first studies to assess the prognostic utility of SII in the context of pharmacologic rhythm control for acute-onset AF, thereby offering a potentially valuable biomarker to guide therapeutic decision-making.

The relationship between inflammation and atrial fibrillation has been increasingly recognized over the past two decades. Inflammatory mechanisms are implicated in both the initiation and maintenance of AF by contributing to atrial electrical and structural remodeling, fibrosis, and oxidative stress [14]. Biomarkers reflecting systemic inflammation—such as C-reactive protein (CRP), interleukin-6 (IL-6), and various cellular indices—have previously been associated with AF recurrence after cardioversion or ablation [15,16,17]. The SII index, calculated as neutrophils × platelets/lymphocytes, integrates multiple aspects of the inflammatory response and has shown prognostic relevance in cardiovascular diseases, including heart failure and myocardial infarction [18,19].

Our findings align with and expand upon prior literature by specifically evaluating SII as a predictor of pharmacologic cardioversion efficacy. The observed inverse relationship between SII and cardioversion success suggests that patients with heightened systemic inflammation may have more advanced atrial remodeling, impaired myocardial electrophysiological responsiveness, or elevated arrhythmogenic substrate burden—factors that may reduce the likelihood of conversion to sinus rhythm with amiodarone alone. Furthermore, lymphopenia and neutrophilia, as components of SII, reflect both immune suppression and innate immune activation, respectively—mechanisms that have been separately linked to arrhythmogenesis [20].

The superior predictive accuracy of left atrial volume index (LAVI) compared to SII (AUC: 0.833 vs. 0.803, respectively) is consistent with its well-established role as a marker of atrial structural remodeling and long-standing pressure overload [21]. Nonetheless, SII maintained significant independent prognostic value in multivariate analysis. These findings suggest that inflammation and structural remodeling are both critical, yet partially non-overlapping, determinants of cardioversion success.

Importantly, the identified SII cut-off of 645.16 yielded a sensitivity and specificity of 75%, indicating strong clinical utility for risk stratification. Incorporating this threshold into pre-cardioversion evaluations could help identify patients who are less likely to respond to pharmacologic therapy, thereby facilitating earlier consideration of alternative strategies such as electrical cardioversion or long-term rhythm control approaches.

Recent evidence has increasingly emphasized the multifactorial nature of cardioversion success in atrial fibrillation, extending beyond arrhythmia duration and pharmacological choice to include atrial substrate characteristics and systemic conditions. Contemporary data suggest that inflammatory status and atrial remodeling are closely intertwined with rhythm-control outcomes, particularly in the early stages of AF management. In this context, recent work has highlighted the importance of integrating clinical, echocardiographic, and biological markers to refine patient selection for cardioversion strategies and improve rhythm-control efficacy [22].

Our findings are consistent with this evolving paradigm, demonstrating that elevated systemic immune-inflammation index (SII) levels are associated with lower rates of successful pharmacological cardioversion. Importantly, given the observational design of the present study and the absence of direct structural or mechanistic assessments—such as atrial fibrosis quantification or advanced imaging—these results should be interpreted as associative rather than causal. Elevated SII may reflect a systemic inflammatory milieu that coexists with more advanced atrial remodeling or electrical instability, thereby reducing responsiveness to antiarrhythmic therapy.

Taken together, the present results support the concept that inflammation-related biomarkers, when interpreted alongside established echocardiographic parameters such as left atrial volume index, may contribute to a more comprehensive risk stratification framework for rhythm-control decision-making in acute-onset AF. Further prospective and mechanistic studies are warranted to clarify the biological pathways linking systemic inflammation with cardioversion outcomes and to determine whether targeted modulation of inflammatory burden can favorably influence rhythm-control success.

From a clinical perspective, SII represents an inexpensive and readily available biomarker that may be used as an adjunctive tool for pre-procedural risk stratification in patients undergoing pharmacological cardioversion. Elevated SII levels may help identify individuals with a lower likelihood of rhythm restoration using antiarrhythmic therapy alone, thereby supporting shared decision-making and consideration of alternative rhythm-control strategies.

This study has several strengths, including its prospective design, homogeneous cohort of patients with acute-onset symptomatic AF, and use of a standardized pharmacologic intervention (intravenous amiodarone). However, several limitations of this study should be acknowledged. First, the study population consisted predominantly of relatively young patients with low CHA_2_DS_2_-VASc scores and limited cardiovascular comorbidity, which may restrict the generalizability of our findings to older patients or those with a higher cardiovascular risk burden. Second, although patients with overt inflammatory conditions such as thyroid dysfunction, myocardial injury, or heart failure were excluded, subclinical infections or chronic inflammatory disorders could not be entirely ruled out and may have influenced SII levels. Third, we did not include other inflammatory biomarkers such as IL-6, which could have provided comparative or additive prognostic information. Fourth, long-term follow-up was not performed, and thus the durability of rhythm control and clinical outcomes beyond hospital discharge remains unknown. Finally, the single-center design and modest sample size further limit external validity.

Future studies should aim to externally validate our findings in larger, multicenter cohorts and assess whether combining SII with echocardiographic and clinical parameters improves the predictive power for cardioversion outcomes. Furthermore, interventional studies could explore whether modulation of systemic inflammation prior to cardioversion—e.g., through corticosteroids, colchicine, or lifestyle interventions—could enhance pharmacologic rhythm control success.

## 5. Conclusions

In patients with new-onset atrial fibrillation, elevated systemic immune-inflammation index levels were independently associated with a lower likelihood of successful pharmacological cardioversion using amiodarone. Given its simplicity and accessibility, SII may serve as a useful adjunctive marker for risk stratification; however, its role should be interpreted within the context of established clinical and echocardiographic parameters.

## Figures and Tables

**Table 1:** Demographic distribution and laboratory findings of all patients.

	Rhythm Control		
	no(24) Mean ± Std.	yes(71) Mean ± Std.	*p*
**Age** (years)	52.29 ± 10.05	54.86 ± 9.66	0.268
**Gender** (female, %)	10 (41.7%)	36 (50.7%)	0.486
**BSA** (m^2^)	1.81 ± 0.11	1.87 ± 0.12	0.024
**LAVI** (mL/m^2^)	38.79 ± 8.60	29.03 ± 5.22	0.000
**CHA_2_DS_2_-VASc Score**	0.92 ± 0.97	0.72 ± 0.83	0.336
**Glucose** (mg/dL)	176.21 ± 64.59	143.66 ± 62.82	0.032
**Total Cholesterol** (mg/dL)	194.79 ± 50.49	192.37 ± 33.14	0.788
**LDL** (mg/dL)	118.00 ± 46.66	114.87 ± 28.47	0.697
**HDL** (mg/dL)	43.75 ± 4.88	41.90 ± 7.64	0.270
**Triglyceride** (mg/dL)	164.83 ± 53.27	172.62 ± 62.89	0.588
**Creatinine** (mg/dL)	0.81 ± 0.15	0.83 ± 0.15	0.651
**IVS Thickness** (mm)	10.71 ± 1.52	10.82 ± 1.43	0.752
**LVEDD** (mm)	47.42 ± 4.32	47.31 ± 3.07	0.895
**LVESD** (mm)	31.21 ± 6.11	30.11 ± 4.40	0.344
**EF** (%)	64.08 ± 2.34	63.04 ± 3.34	0.162
**HR** (bpm)	160.00 ± 9.96	159.80 ± 10.45	0.936
**Hb** (g/dL)	13.47 ± 2.09	13.75 ± 2.06	0.571
**WBC** (×10^3^/µL)	9.32 ± 1.79	8.21 ± 2.14	0.025
**Neutrophil** (×10^3^/µL)	6.31 ± 1.97	4.81 ± 1.74	0.001
**Lymphocyte** (×10^3^/µL)	1.87 ± 0.68	2.43 ± 0.71	0.001
**Platelet** (×10^3^/µL)	282.46 ± 54.40	276.42 ± 56.98	0.651
**CRP** (mg/L)	6.89 ± 3.15	7.89 ± 3.08	0.174
**SII**	1108.17 ± 730.45	598.76 ± 369.45	0.003

BSA, Body Surface Area; LAVI, Left Atrial Volume Index; IVS, İnterventricular Septum; EF, Ejection Fraction; HR, Heart Rate; Hb, Hemoglobin; WBC, White Blood Cell; CRP, C-Reactive Protein; SII, Systemic Immune-Inflammation Index.

**Table 2:** Multiple logistic regression analysis showing independent predictors of pharmacological cardioversion success in patients with acute-onset symptomatic AF.

Variable (Per 1 SD Increase)		Univariate Analysis			Multivariate Analysis	
	B	OR (95% CI)	*p* Value	B	OR (95% CI)	*p* Value
**LAVI (standardized)**	−1.464	0.23 (0.12–0.45)	<0.001	−1.708	**0.18 (0.08–0.42)**	<0.001
WBC (standardized)	−0.558	0.57 (0.35–0.94)	0.029	0.102	1.11 (0.42–2.92)	0.836
**SII (standardized)**	−1.058	0.35 (0.19–0.64)	0.001	−1.519	**0.22 (0.08–0.58)**	0.003
Glucose (standardized)	−0.466	0.63 (0.40–0.98)	0.042	−0.666	0.51 (0.26–1.01)	0.050
BSA (standardized)	0.588	1.80 (1.07–3.03)	0.027	0.219	1.25 (0.56–2.77)	0.587

Adjusted for BSA, WBC, Glucose, SII, LAVI. CI, confidence interval; OR, Odds ratio; BSA: body surface area, WBC: white blood cell, LAVI: Left Atrial Volume Index, SII: systemic immune inflammatory index.

## Data Availability

All needed data can be obtained from the corresponding authors.
